# Exoproteomic Study and Transcriptional Responses of Laccase and Ligninolytic Peroxidase Genes of White-Rot Fungus *Trametes hirsuta* LE-BIN 072 Grown in the Presence of Monolignol-Related Phenolic Compounds

**DOI:** 10.3390/ijms241713115

**Published:** 2023-08-23

**Authors:** Konstantin V. Moiseenko, Olga A. Glazunova, Olga S. Savinova, Tatyana V. Fedorova

**Affiliations:** A. N. Bach Institute of Biochemistry, Research Center of Biotechnology, Russian Academy of Sciences, Leninsky Ave. 33/2, Moscow 119071, Russia; mr.moiseenko@gmail.com (K.V.M.); savinova_os@rambler.ru (O.S.S.); fedorova_tv@mail.ru (T.V.F.)

**Keywords:** *Trametes hirsuta*, white-rot fungi, aromatic compounds, monolignols, laccases, ligninolytic peroxidases, exoproteome, transcriptional response

## Abstract

Being an abundant renewable source of aromatic compounds, lignin is an important component of future bio-based economy. Currently, biotechnological processing of lignin through low molecular weight compounds is one of the conceptually promising ways for its valorization. To obtain lignin fragments suitable for further inclusion into microbial metabolism, it is proposed to use a ligninolytic system of white-rot fungi, which mainly comprises laccases and peroxidases. However, laccase and peroxidase genes are almost always represented by many non-allelic copies that form multigene families within the genome of white-rot fungi, and the contributions of exact family members to the overall process of lignin degradation has not yet been determined. In this article, the response of the *Trametes hirsuta* LE-BIN 072 ligninolytic system to the presence of various monolignol-related phenolic compounds (veratryl alcohol, *p*-coumaric acid, vanillic acid, and syringic acid) in culture media was monitored at the level of gene transcription and protein secretion. By showing which isozymes contribute to the overall functioning of the ligninolytic system of the *T. hirsuta* LE-BIN 072, the data obtained in this study will greatly contribute to the possible application of this fungus and its ligninolytic enzymes in lignin depolymerization processes.

## 1. Introduction

Lignin valorization has been a recurring theme in chemistry and biotechnology for over a century. As an essential part of plant cell walls, lignin is the second most abundant biopolymer on Earth after cellulose [1]. The pulp and paper industry annually produces tens of millions of tons of lignin, which are mostly burned to generate thermal energy [2]. Difficulty of lignin valorization stems from its complex chemical nature. Lignin is a collection of highly heterogeneous irregular polymers, consisting mainly of three phenylpropanoid units—guaiacyl, syringyl, and 4-hydroxyphenyl [3].

To date, the depolymerization of lignin into low molecular weight products is one of the promising methods for its valorization. Some products of lignin depolymerization can be valuable chemicals in their own right. For example, 15% of the world’s vanillin is currently produced by the oxidation of lignosulfonate or kraft-lignin [4]. Alternatively, products of lignin depolymerization can be further processed using microbial cell factories [5,6,7]. The current state of knowledge about the bacterial metabolism of aromatic compounds, together with the scientific and technical development of synthetic biology, allows the development of highly specific processes for the bioconversion of lignin depolymerization products into aromatic platform chemicals, biologically active natural products, plastic precursors, and many others [8,9].

Since all current commercial-scale lignin depolymerization methods (i.e., thermal, chemical, and microwave-assisted depolymerization) require high energy input and harsh conditions, the search for an alternative cost-efficient lignin depolymerization process is an active area of research [10]. One of the promising methods for lignin depolymerization is the use of an enzymatic ligninolytic complex secreted by a special class of wood-rotting fungi—white-rot fungi. These fungi are the only organisms known to be able to utilize lignin efficiently and therefore have a unique ability to depolymerize it [11,12,13]. The group of white-rot fungi is dominated by basidiomycetes of the order Polyporales, of which the representatives of the Core Polyporoid clade—such as *Trametes* spp., *Pycnoporus* spp. and *Polyporus* spp.—are the best known [14,15].

Currently, two groups of enzymes responsible for the oxidative lignin depolymerization by white-rot fungi were identified: laccases and ligninolytic peroxidases [16]. Laccases are copper-containing enzymes that catalyze oxidation of a wide range of substituted phenolic compounds with the simultaneous reduction of oxygen to water [17,18]. It is believed that laccases can directly oxidize only phenolic lignin structures; however, the oxidation of non-phenolic lignin structures is still possible with the help of low-molecular-weight mediator compounds [19]. The ligninolytic peroxidases (hereinafter referred to as peroxidases) are a group of heme-containing enzymes that use H_2_O_2_ as an electron acceptor for the oxidation of lignin and lignin-like compounds [20]. The peroxidases are subdivided into lignin, manganese, and versatile peroxidases based on their substrate specificity [21,22]. Lignin peroxidases (LiP) are capable of oxidizing phenolic and, presumably, non-phenolic lignin structures [23]. Manganese peroxidases (MnP) can oxidize manganese ions, which, in turn, can oxidize the phenolic lignin structures. In addition, some manganese peroxidases can directly oxidize lignin-like substrates through yet unknown mechanisms [24,25]. Versatile peroxidases (VP), as their name suggests, possess catalytic features of both lignin and manganese peroxidases [23].

In the genomes of white-rot fungi, laccase and peroxidase genes are almost always represented by many non-allelic copies that form multigene families [26,27,28,29]. Thus, each fungus can potentially secrete a number of different laccase and peroxidase isozymes (i.e., enzymes expressed from different genes), and the contributions of different isozymes to the overall processes of lignin degradation have not yet been determined. Although there are plenty of works devoted to the response of fungal ligninolytic systems to wood and wood-related components (e.g., cellulose and lignin), only several studies have been conducted on the transcriptional response of white-rot fungi to monolignol-related phenolic compounds (MRPCs), which could be formed during the process of lignin degradation [30,31,32,33]. Moreover, to date there is the only one study containing data on the exoproteomic response of the white-rot fungus to MRPCs [34,35].

Belonging to the Core Polyporoid clade, *Trametes hirsuta* (Wulfen) Lloyd 1924 is a typical representative of white-rot fungi. The genome of *T. hirsuta* contains seven laccase and eighteen peroxidase (nine LiP, seven MnP, and two VP) encoding genes [36,37,38]. In this article, the response of the *T. hirsuta* LE-BIN 072 ligninolytic system to the presence of various MRPCs in culture media was monitored at the level of gene transcription and protein secretion. In addition, previous exoproteomic studies of *T. hirsuta* cultivated on various media were summarized.

## 2. Results and Discussion

### 2.1. Growth and Overall Oxidative Activity

**Figure 1:**
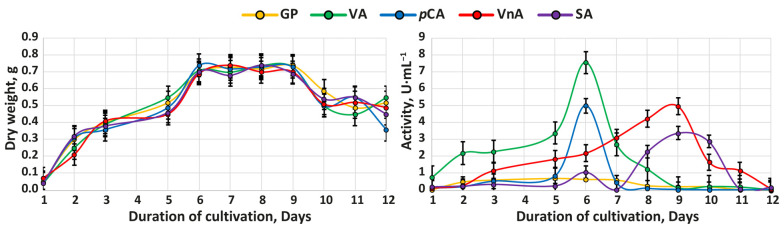
The dynamics of biomass accumulation and changes in the overall oxidative activity of the cultural broth during cultivation of *T. hirsuta* 072 on the control glucose-peptone (GP) medium and GP media supplemented with veratryl alcohol (VA, 2 mM), *p*-coumaric acid (*p*CA, 0.4 mM), vanillic acid (VnA, 0.2 mM), or syringic acid (SA, 0.2 mM). The bars on the graphs represent the mean ± standard deviation.

The dynamics of biomass accumulation and changes in the overall oxidative activity of the cultural broth during cultivation of *T. hirsuta* LE-BIN 072 (hereinafter referred to as *T. hirsuta* 072) on the control GP medium and GP media supplemented with four MRPCs—veratryl alcohol (VA), *p*-coumaric acid (*p*CA), vanillic acid (VnA), or syringic acid (SA)—are presented in Figure 1. All of these compounds can be regarded as structural units of lignin and can be formed during its chemical or biological degradation [39,40,41].

During cultivation, *T. hirsuta* 072 showed the same dynamics of biomass accumulation on all the studied media: the logarithmic growth phase was observed between the first and sixth days of cultivation; the stationary phase was observed between the sixth and ninth days of cultivation; and slow biomass decline was observed after the ninth day of cultivation. For all cultivations, the profile of the overall oxidative activity has a pronounced maximum. For the VA- and *p*CA-containing media, the maximal activity was observed at the sixth day of cultivation, and for the VnA- and SA-containing media—at the ninth day of cultivation.

Existing data on the cultivation of various wood-degrading fungi in the presence of aromatic compounds show that these compounds can have a growth-inhibiting effect even at low concentrations. In particular, it was shown that some MRPCs can exhibit strong antifungal properties depending on the length of the CH chain attached to the benzene ring and the presence of conjugated double bonds, carbonyl or carboxyl functional groups in this chain [42,43]. Generally, compounds based on guaiacyl and *p*-hydroxyphenyl lignin units demonstrated higher antifungal properties than compounds based on syringyl units [44]. In addition, compounds with methylated phenolic hydroxyl and carboxyl groups showed a more pronounced growth-inhibiting effect on the fungal mycelium compared to non-methylated ones [45].

From published data, it also can be hypothesized that among wood-degrading fungi, those with a more developed ligninolytic system are more resistant to growth inhibition by MRPCs. Accordingly, brown-rot fungi were reported to be more sensitive to the growth-inhibition compared to white-rot fungi [44,45,46,47]. At the same time, among white rot fungi, the most insensitive to MRPCs were those that have a more aggressive strategy of wood colonization (i.e., primary colonizers) and, therefore, have a more developed ligninolytic system [48]. For example, *Dichomitus squalens*, an efficient wood-degrading white-rot fungus, did not show any growth inhibition when cultivated with 10 different MRPCs [30].

The present study also substantiates the insensitivity of primary colonizing white-rot fungi to MRPCs, since none of the tested MRPCs had a significant inhibitory effect on the growth of *T. hirsuta* 072 (Figure 1). Moreover, it was previously shown that *T. hirsuta* 072 does not exhibit growth inhibition when cultivated in the presence of kraft lignin and, consequently, in the presence of all derivatives formed as a result of kraft lignin oxidative depolymerization by the ligninolytic system of the fungus [38,49]. However, there are not enough data to comprehensively generalize about the insensitivity of primary colonizing white-rot fungi to MRPCs, and additional experiments with various white-rot fungi are required.

### 2.2. Exoproteomic Study

**Figure 2:**
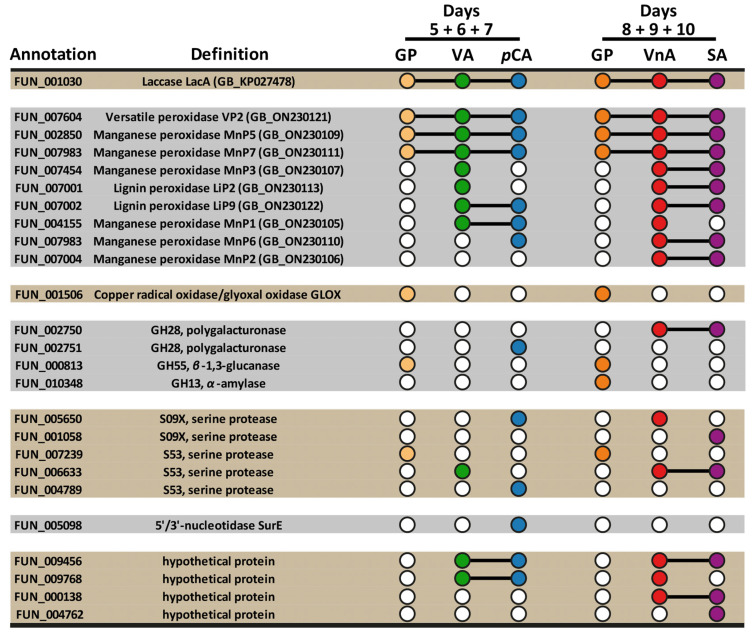
The data on the composition of the *T. hirsuta* 072 exoproteomes obtained during its cultivation on the control glucose-peptone (GP) medium and GP medium supplemented with veratryl alcohol (VA), *p*-coumaric acid (*p*CA), vanillic acid (VnA), or syringic acid (SA). The phrase like “days 5 + 6 + 7” means that culture broth from days 5, 6, and 7 of cultivation were pooled together. The annotation of the *T. hirsuta* 072 genome used in this article can be found in the Supplementary Materials section (Supplementary Files S1–S4).

For the exoproteomic study, the samples of cultural broth were collected around those time points where the overall oxidative activity was the highest (Figure 1)—at the fifth, sixth, and seventh days of cultivation for the media supplemented with VA and *p*CA and at the eighth, ninth, and tenth days of cultivation for the media supplemented with VnA and SA. For each medium, the collected samples were pooled together, and proteins were extracted. The data on the composition of the *T. hirsuta* 072 exoproteomes obtained on the different cultivation media are summarized in Figure 2.

The majority of enzymes secreted by *T. hirsuta* 072 belonged to the fungal ligninolytic complex and were represented by one laccase isozyme (LacA) and nine isozymes of ligninolytic peroxidases—one versatile peroxidase (VP2), six manganese peroxidases (MnP1, MnP2, MnP3, MnP5, MnP6, and MnP7), and two lignin peroxidases (LiP2 and LiP9). Additionally, *T. hirsuta* 072 secreted four glycoside hydrolases (from GH13, GH28, and GH55 families), five serine proteases (from S09X and S53 families), one glyoxal oxidase, one nucleotidase, and four proteins with unknown function. All secreted proteins had proper signal peptides and were therefore secreted by the classical secretory pathway.

The obtained data confirm the hypothesis put forward earlier that *lacA* of *T. hirsuta* 072 is a gene that is expressed by default (i.e., “default on”) [50]. As in the current study, in the studies where *T. hirsuta* 072 was cultivated on the media containing CuSO_4_, kraft lignin, synthetic dye (i.e., bromocresol green), lignocellulose and sawdust of various wood (i.e., alder, birch, or pine), LacA isozyme was always present in the fungal exoproteome (Supplementary Table S1) [38,49,51,52]. In addition, previous studies suggest that *lacA* may be regulated by both repressive and inductive mechanisms. While on the copper-containing medium the amount of LacA in the fungal exoproteome drastically increased [51], on the medium supplemented with kraft-lignin, LacA was undetectable already after the third day of cultivation [49]. As for other laccase isozymes, the only other isozyme ever found in the exoproteome of *T. hirsuta* 072 was LacC, which was detected in a small amount during cultivation on the lignocellulose-containing medium [51].

Using previously obtained data about orthologous groups of laccases and peroxidases from different fungi [48,53], it could be concluded that orthologues of LacA isozyme from *T. hirsuta* 072 are the most abundant laccase isozymes secreted under various conditions by fungi from *Trametes* and, closely related to it, *Pycnoporus* genera. For example, LacA orthologues were found in exoproteomes of three *Pycnoporus* spp. cultivated on control maltose-containing medium as well as on media supplemented with cellulose, wheat straw, aspen chips, and grass biomass [54,55]. In addition, LacA orthologue was secreted by *Trametes versicolor* A1-ATF during its cultivation on aspen wafers [56]. However, in a study of the *T. versicolor* FP-101664 exoproteomic response to MRPCs, no laccase isozymes were detected in exoproteomes during cultivations with either syringic or 4-hydroxybenzoic acids [34].

With respect to the secretion of peroxidases, it can be concluded that for *T. hirsuta* 072, MRCPs induce secretion of broader spectrum of peroxidase isozymes than lignin itself. In the current study, *T. hirsuta* 072 secreted from six to nine peroxidase isozymes on all media supplemented with MRCPs, while in the previous studies lignin induced secretion from two to six peroxidase isozymes at different stages of cultivation (Supplementary Table S1) [38,49]. Moreover, it was previously reported that in GP medium supplemented with bromocresol green dye, which contains two substituted phenolic units resembling MRCPs, *T. hirsuta* 072 secreted seven peroxidase isozymes [38].

Possibly, the ability to simultaneously secrete many peroxidase isozymes is a distinctive feature of *T. hirsuta* species. Similarly with *T. hirsuta* 072, the *T. hirsuta* strain X-13 cultivated on lignin-containing media was reported to secrete up to six peroxidase isozymes [57], while the related fungi from *Trametes* and *Pycnoporus* genera were reported to secret only from one to four peroxidase isozymes even in wood-containing media [34,54,55,58,59]. The only one reported exception from the above is *T. versicolor* strain A1-ATF, which secreted from five to nine peroxidase isozymes when cultivated on aspen wafers [56,60].

All peroxidases identified in the exoproteomes of *T. hirsuta* 072 during this study could generally be divided into two groups. The first group of peroxidases contained isozymes that were present in all exoproteomes—VP2, MnP5, and MnP7. Interestingly, these three peroxidase isozymes were previously observed in almost all exoproteomes of *T. hirsuta* 072 ever published, including those obtained on kraft lignin, lignocellulose, bromocresol green, and sawdust of various wood (Supplementary Table S1) [38,49,51,52]. The only notable exception was the absence of MnP7 in exoproteomes obtained on the wood-containing media [52]. Possibly, expression of this isozyme was inhibited by the presence of cellulose, hemicellulose, or wood extractives.

The second group of peroxidases included isozymes that were present on media containing MRPCs but were absent on control GP media—LiP2, LiP9, MnP1, MnP2, MnP3, and MnP6. Among these peroxidases, each isozyme was secreted on the several MRPC-containing media; therefore, there were no peroxidase isozymes specifically secreted in response to the presence of certain MRPC in the medium. It can be noted that in this study, LiP9 was secreted on all MRPCs-containing media, and LiP2—on the media supplemented with VA, VnA, and SA; however, during previous cultivations of *T. hirsuta* 072 on lignin and sawdust of various wood, the secretion of these isozymes was either absent or was observed in negligible amount only at the late stages of cultivation [49,52]. Therefore, it can be assumed that *T. hirsuta* 072 secretes LiP2 and LiP9 mainly for the detoxification of small MRCPs, and not for the depolymerization of the lignin macromolecule. A similar assumption can be made for MnP1. In this study, MnP1 was secreted on two of the three MRCPs tested; however, this isozyme was previously encountered only once on lignin at the late stage of cultivation, when a significant amount of MRCPs accumulated in the medium [49]. Unfortunately, for MnP2, MnP3, and MnP6, no obvious patterns of secretion can be noted so far.

As mentioned above, MnP5 is one of the most abundant peroxidase isozymes in exoproteoms of *T. hirsuta* 072 regardless of cultivation conditions; however, this is not the case of other *Trametes* and *Pycnoporus* spp. Notably, the exoproteome of another *T. hirsuta* strain X-13 did not contain MnP5 isozyme at all [57]. The only exoproteome in which MnP5 orthologue was found was the exoproteome of *T. versicolor* cultivated on media containing syringic acid [34]. Thus, the “default on” secretion of MnP5 isozyme may be an individual feature of the *T. hirsuta* 072 strain.

Among other peroxidase isozymes, which orthologues were previously encountered in the exoproteomes of fungi closely related to *T. hirsuta*, LiP9, VP2, and MnP7 are especially worth emphasizing. The orthologue of LiP9 was previously observed only in the exoproteome of *T. versicolor* A1-ATF cultivated on aspen wafers [56]. As for the orthologues of VP2 and MnP7 isozymes of *T. hirsuta* 072, these isozymes were previously reported in exoproteomes of *Trametes* and *Pycnoporus* spp. in various conditions including media supplemented with cellulose, lignin, lignocellulose, and copper [34,55,56,57,58,59,60].

### 2.3. Transcription of Laccases and Ligninolytic Peroxidases

**Figure 3:**
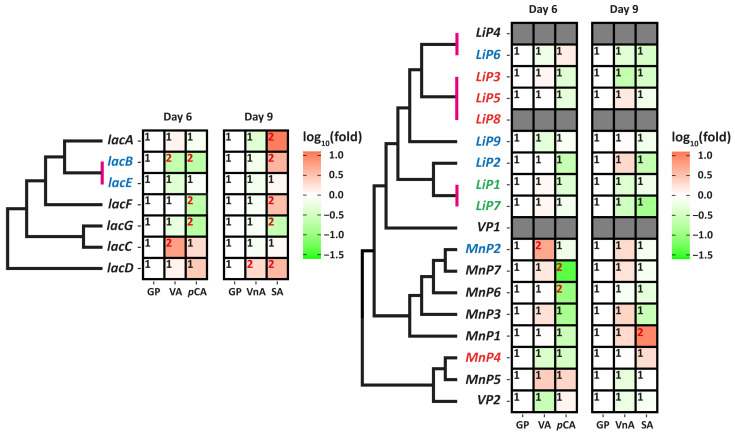
The data on the transcription of laccase and ligninolytic peroxidase genes of *T. hirsuta* 072 during its cultivation on the control glucose-peptone (GP) medium and GP media supplemented with veratryl alcohol (VA), *p*-coumaric acid (*p*CA), vanillic acid (VnA), or syringic acid (SA). The fold change in transcription is expressed relative to the control GP medium. Within each row, cells with different superscripts represent significantly different levels of transcription (*p* < 0.05). Each heat-map is complemented by a phylogenetic tree on the left, and those genes that originated from species-specific duplications (i.e., inparalogues) are united by vertical-colored bars. On each tree, genes of the same color (except for black) form clusters with each other on the chromosomes.

For the study of the transcription of laccase and peroxidase genes, the samples of fungal mycelium were collected at the time points where the overall oxidative activity was the highest (Figure 1)—at day 6 of cultivation for the media supplemented with VA and *p*CA and at day 9 of cultivation for the media supplemented with VnA and SA. The data on the gene transcription are summarized in Figure 3.

For both laccase and peroxidase genes, changes in the level of transcription on media supplemented with MRCPs did not differ from those on the control GP medium by more than 10-fold. Transcription of all laccase genes and 15 peroxidase genes was detected on all studied media; three peroxidase genes (*LiP4*, *LiP8*, and *VP1*) were not transcribed on any medium. No correlation was found between gene transcription patterns and their phylogenetic relationships. In addition, genes located in close proximity to each other (i.e., forming a cluster on the chromosome) did not show similar transcriptional patterns.

In general, the transcription patterns of laccase genes on *p*CA and SA media observed in this study are similar to those previously seen after cultivation of *T. hirsuta* 072 on MRCPs for three days, although they are not so pronounced [50]. For ***p*CA** medium after the third day of cultivation, it was reported [50] that transcription of all laccase genes was strongly suppressed: *lacF*, *lacB,* and *lacG* by 125–260-fold; *lacC*, *lacD,* and *lacE* by 30-fold; and *lacA* by 8-fold. In the current experiment, transcription of *lacB*, *lacG,* and *lacB* on the sixth day of cultivation on *p*CA was also suppressed from three- to five-fold, and transcription of the other laccase genes was not significantly different (*p* > 0.05) from that on the control GP medium. For SA medium after the third day of cultivation, it was reported [50] that transcription of *lacC* and *lacB* was induced by approximately six-fold, transcription of *lacF* was induced by 36-fold, and transcription of *lacG* was undetectable. In the current study transcription of *lacB*, *lacF* was also increased by approximately four-fold and transcription of *lacG* was suppressed by five-fold. Additionally, transcription of *lacA* and *lacD* was increased by 4 ± 1- and 11 ± 3-fold, respectively. For VA and VnA, the transcriptional patterns found in this study were completely different from those previously observed after cultivation of *T. hirsuta* 072 on this media for three days [50].

In the case of peroxidase genes, the observed transcription pattern was different from that previously reported for *T. hirsuta* 072 cultivated in the presence of bromocresol green and kraft lignin [38]. Whereas bromocresol green dramatically suppressed the transcription of all peroxidase genes, kraft lignin strongly stimulated the transcription of seven peroxidase genes and at the same time completely suppressed (i.e., transcription was completely absent) the transcription of others. In the current study, MRCPs stimulated the transcription of some peroxidase genes and suppressed others, but none of the peroxidase genes initially transcribed on the control GP medium were suppressed completely. Perhaps such a “mixed” transcription pattern is due to the fact that on the one hand, MRCPs are structural units of lignin, and on the other hand, they are xenobiotics similar to bromocresol green.

Comparing the results of this study with those obtained for other polypore fungi, it can be assumed that there is a significant time lag between the increase in laccase and peroxidase gene transcription and secretion of the corresponding isozymes into the medium. While it was shown that in principle transcription levels of laccase and peroxidase genes can vary by 2–3 orders of magnitude [32,61], the difference between gene expression during growth on the control medium and the medium with inducers after several days of cultivation (when the secretion of enzymes becomes apparent according to the activity profile or proteomic data) usually did not exceeded 10–30-fold [30,33,62]. In addition, several experiments have shown that laccase transcription can be increased 100–500-fold within hours after the addition of inducing compounds and become negligible when isozyme secretion becomes detectable [32,33,50]. In our study, signs of such a process are visible for media supplemented with *p*CA and SA, where the transcription patterns on days 6 and 9 generally coincided with that earlier reported on day 5 [50], but the increase in transcription relative to the control GP medium was significantly lower. The situation with the discrepancy of transcription and secretion can be common for all enzymes secreted by polypore fungi. This was most clearly supported by the works devoted to the *D. squalens* [63,64] and *Pycnoporus* spp. (*P. coccineus*, *P. sanguineus,* and *P. cinnabarinus*) [55], where only approximately 50% of all exoenzymes (i.e., that could be secreted) whose genes were transcribed were found in exoproteomes.

## 3. Materials and Methods

### 3.1. Fungal Strain and Culture Conditions

The strain of the white-rot fungus *T. hirsuta* LE-BIN 072 was obtained from the Komarov Botanical Institute Basidiomycetes Culture Collection (LE-BIN; St. Petersburg, Russia). After receiving from the collection, the strain was stored at a temperature of +4 °C on slant wort agar (20% *v*/*v* of 12% wort; 2% *w*/*v* of agar). The fungal culture was stored without reseeding from six months to one year.

To obtain starting inoculum, fungal mycelium was subcultured from slant wort agar onto Petri dishes (d = 90 mm) containing wort agar of the same composition. Petri dishes were cultivated in the dark at 28 °C until the surface of the dish was completely covered with mycelium. Then, pieces of mycelium from the Petri dish (~1/4 of the dish) were added to 750 mL conical flasks containing ceramic beads and 200 mL of glucose peptone (GP) medium. The seeded flasks were incubated in the dark at 26–28 °C for 7 days. Before further inoculation on the studied media, the mycelium of the fungus was crushed with ceramic beads at 250 RPM for 15 min. Inoculation was carried out with 20 mL of the resulting suspension.

The composition of the GP medium was as follows (g∙L^−1^): glucose—10.0; peptone—3.0; KH_2_PO_4_—0.6; ZnSO_4_ × 7H_2_O—0.001; K_2_HPO_4_—0.4; FeSO_4_ × 7H_2_O—0.0005; MnSO_4_—0.05; MgSO_4_ × 7H_2_O—0.5; CaCl_2_—0.25. The medium was autoclaved at 120 °C and 1 atm for 30 min.

For cultivation of *T. hirsuta* 072 in the presence of MRPCs, the 200 µL of MRPC solutions in ethanol were added to the autoclaved GP medium. The final concentrations of MRPCs were as follows: veratryl alcohol—2 mM, *p*-coumaric acid—0.4 mM, vanillic acid—0.2 mM, and syringic acid—0.2 mM. The cultivation was carried out with constant agitation (180 RPM) at a temperature of 25 °C in a dark aerated chamber of an orbital shaker Innova 44 (New Brunswick Scientific, Edison, NJ, USA).

### 3.2. Growth Rate, Overall Oxidative Activity Assay, Exoproteomic and Transcriptional Studies

To measure the dry weight, samples of the mycelium were dried at 105 °C to a constant weight, and air-dry biomass was determined by the gravimetric method.

The overall oxidative activity was measured using ABTS as a substrate by monitoring the increase in absorbance at 436 nm for 3 min. The substance detected in the reaction was the oxidation product of ABTS (ε_436_ = 29,500 M^−1^ cm^−1^). The enzymatic reaction was carried out in 2 mL of 0.1 M sodium acetate buffer (pH 4.5), 1 mM ABTS, and the required amount of culture liquids to reliably determine the initial reaction rates. One unit of enzymatic activity was expressed as μM of product formed per min.

The exoproteome extraction, sample preparation, two-dimensional gel electrophoresis (2-DE), MALDI-TOF/TOF MS/MS analysis, and data processing were performed as previously described in Moiseenko et al. [49].

The RNA extraction, reverse transcription, and qPCR analysis for laccase genes were performed as described in Moiseenko et al. [50]. For the qPCR analysis of transcription of peroxidase genes, the primers and TaqMan probes previously described by Vasina et al. [38] were used, and the qPCR cycling conditions were as follows: initial denaturation at 95 °C for 10 min; 40 amplification cycles (30 s at 95 °C, 1 min at 60 °C).

### 3.3. Statistical Data Manipulations

All experiments were performed in three biological replicates, and data are presented as mean ± standard deviation when appropriate. All statistical comparisons were firstly performed using a one-way ANOVA omnibus *F*-test. When a significant (*p* < 0.05) value of *F*-statistics was found, differences between means were evaluated using Tukey’s HSD (honestly significant difference) multiple comparison test (*p* < 0.05).

## 4. Conclusions

In summary, the current investigation highlighted that as primary colonizing white-rot fungus, *T. hirsuta* 072 can grow not only in presence of lignin but in the presence of monolignol-related phenolic compounds (MRPCs), which can be toxic in general for other fungi. It was demonstrated that for *T. hirsuta* 072, MRCPs induce secretion of broader spectrum of ligninolytic peroxidase isozymes than lignin. The MRPCs can trigger expression of such peroxidase isozymes as MnP1, MnP2, MnP3, MnP6, LiP2, and LiP9, among which the secretion of LiP2 was never observed in previous studies. In addition, it was shown that such peroxidase isozymes as MnP5, MnP7, and VP2 were always secreted by *T. hirsuta* 072 regardless of the presence of MRPCs in the medium. It has been suggested that the simultaneous secretion of many peroxidase isozymes is a hallmark of *T. hirsuta* as a species. In addition, it was shown that it is unusual for fungi closely related to *T. hirsuta* (i.e., from *Trametes* and *Pycnoporus* genera) to secrete orthologues of the MnP5 isozymes that is always secreted by *T. hirsuta* 072 by default. Surprisingly, MRPCs did not induce secretion of laccase isozymes other than LacA, which *T. hirsuta* 072 almost always expresses by default. Additionally, it was again highlighted that in white-rot fungi, gene transcription, protein secretion, and activity observed in cultural broth do not strictly correlate with each other. This important observation must be always accounted for in further investigations of white-rot fungi in which measurements are taken at only one of the mentioned biological levels.

## Data Availability

The data presented in this study are available within the article.

## Supplementary Materials

The supporting information can be downloaded at: https://www.mdpi.com/article/10.3390/ijms241713115/s1.
